# Advancing Type 2 Diabetes Prevention through Text-Messaging Interventions: A Narrative Review

**DOI:** 10.1007/s11892-024-01568-w

**Published:** 2024-12-04

**Authors:** Taynara Formagini, Christopher J. Gonzalez, Julie Dias, Elva M. Arredondo, Eric Hekler, Matthew J. O’Brien

**Affiliations:** 1 Department of Family Medicine, University of California San Diego, 9500 Gilman Dr., La Jolla, San Diego, CA 92093 USA; 2 Division of General Internal Medicine, Weill Cornell Medicine, New York, NY USA; 3 Department of Biological Sciences, University of Central Florida, Orlando, FL USA; 4 Psychology Department, San Diego State University, San Diego, CA USA; 5 Herbert Wertheim School of Public Health & Human Longevity Science, University of California San Diego, San Diego, CA USA; 6 Department of Medicine, Division of General Internal Medicine and Geriatrics, Northwestern University Feinberg School of Medicine, Chicago, IL USA

**Keywords:** Type 2 diabetes prevention, Diabetes Prevention Program (DPP), Text messaging, Narrative review

## Abstract

**Purpose of Review:**

Text-messaging interventions effectively prevent and manage numerous health conditions. This scoping review evaluates recent literature on text-messaging interventions focused on diabetes prevention, highlighting their development, associated outcomes, reach, and potential sustainability.

**Recent Findings:**

A total of 28 studies met eligibility criteria and were included in this review. Text-messaging was often used as a primary intervention method, focusing on promoting weight loss through physical activity and dietary changes. Studies also explored hybrid approaches integrating text-messaging with in-person sessions or other digital platforms. Intervention development involved multi-phase content creation, often leveraging established diabetes prevention curricula. Studies generally reported high feasibility and acceptability, although effectiveness was mixed. Cost-effectiveness comparisons favored text-messaging over traditional in-person programs. Implementation strategies aligned interventions with existing healthcare workflows, facilitating scalability and integration into routine care practices.

**Summary:**

Text-messaging interventions demonstrate considerable promise but require further refinement to ensure their effectiveness, particularly in enhancing participant engagement to ensure effectiveness and sustainability. Future research should focus on refining intervention content, integrating interactive features, and expanding cost-effectiveness evaluations to support broader implementation in real-world settings.

**Supplementary Information:**

The online version contains supplementary material available at 10.1007/s11892-024-01568-w.

## Introduction

Type 2 diabetes (T2DM) imposes significant public health and economic burdens globally, but substantial progress has been made in its prevention over the past two decades. Multiple randomized clinical trials, including the landmark U.S. Diabetes Prevention Program (DPP), have demonstrated that intensive lifestyle interventions targeting modest weight loss can reduce the risk of developing T2DM [1–4]. Subsequent translational trials with comparable lifestyle interventions in real-world settings replicated similar T2DM risk reduction [5–7]. These findings led to the establishment of the National Diabetes Prevention Program (National DPP) in 2010, which translated the DPP into community settings across the U.S [8]. Since its inception, the National DPP has developed a network of over 1,500 providers delivering the program, achieving clinically significant outcomes [9, 10]. Medicare and selected commercial health insurance plans now offer reimbursement for the National DPP, presenting an opportunity to sustain the program and expand its reach [11].

Despite these advancements, there remains a need to increase the reach and sustainability of T2DM prevention services and to ensure equitable access both in the U.S. and globally [12]. Fewer than 1% of the 97 million Americans with prediabetes have enrolled in the National DPP [13, 14]. This may be due to limited awareness of T2DM risk, as only 15% of those with prediabetes in the U.S. report awareness of the condition [15]. However, the DPP’s high-intensity and weekly in-person requirements may also restrict participation. Among those who do participate, attrition rates are high, particularly among participants from racial and ethnic minority groups [16, 17]. Current reimbursement payments often do not cover the full cost of the program, hindering its sustainability [18, 19]. Internationally, similar challenges persist, particularly in low and middle-income countries [20–23].

There has been a growing interest in alternative delivery methods to expand the reach and ensure sustained implementation of T2DM prevention interventions. Since 2015, the CDC has approved delivery of the National DPP via video conferencing [24]. Capitalizing on the widespread growth of internet access, smartphone ownership, and text-messaging utilization, interventions using these technologies have also emerged [25, 26]. Several reviews have evaluated digital interventions for T2DM prevention, presenting promising feasibility, acceptability, and efficacy [27–35]. However, the heterogeneity of these technologies poses challenges in determining the most effective strategies. Additionally, there is still limited understanding of how these programs are being implemented in real-world settings.

Text-messaging has emerged as a compelling potential strategy for delivering T2DM prevention content, given its demonstrated effectiveness in promoting diverse healthy behaviors [36–40]. Unlike other digital methods, text-messaging enables access for continuous engagement according to participants’ interests and availability [41]. Further, text-messaging does not require advanced technological skills, facilitating participation for individuals with limited literacy [42, 43]. Additionally, it is highly scalable, given the ubiquitous use and ownership of mobile phones compared to computers [44]. Lastly, text-messaging is potentially less costly than more sophisticated digital approaches, which may facilitate implementation in resource-limited settings [45].

A 2022 scoping review examined the use of text-messaging and other digital technology prompts in T2DM prevention, finding mixed evidence on their effectiveness in behavioral outcomes and T2DM incidence [46]. Given rapid advancements in this field, our narrative review aims to critically evaluate recent literature focused on T2DM prevention interventions delivered through text-messaging in the past five years. We examine how text-messaging may promote the reach and sustainability of T2DM prevention content, considering the following factors: program development, feasibility and acceptability, effectiveness, and costs. Additionally, we identify current gaps, propose future research directions, and discuss health equity considerations in developing these interventions.

## Methods

### Search Strategy and Selection Criteria

In April 2024, we conducted a literature search on PubMed, Scopus, and CINAHL to identify relevant studies. Our search methodology on PubMed employed a combination of keywords using Boolean operators and Medical Subject Headings (MeSH). Specifically, we utilized the following search strategies: a) ‘Text Messaging’ [Mesh] and ‘Prediabetic State’ [MeSH], b) ‘Text Messaging’ [MeSH] and ‘diabetes prevention’ [keyword], and c) ‘text-message’, ‘text message’, ‘text-messaging’, ‘texting’, ‘text messages’ and ‘diabetes prevention’ [keywords]. We adopted a comparable search strategy for other databases with the following exceptions: In Scopus, we confined the search to article titles, abstracts, and keywords. In CINAHL, we included the term ‘text-messaging or SMS’ as it corresponds to the search options provided by the database.

Studies were selected based on the following inclusion criteria: (1) Interventions explicitly focused on T2DM prevention. (2) Use of mobile phone text-messaging to deliver the intervention, either exclusively or in combination with other components. For interventions incorporating additional components, text-messaging was required to be a substantive component with specific data on its feasibility, acceptability, or effectiveness. (3) Targeting individuals at risk of T2DM, including those with overweight/obesity, hyperglycemia as measured by A1c, fasting plasma glucose, or oral glucose tolerance test, as well as women with a history of gestational diabetes. Interventions solely targeting participants based on weight status were included only if the study explicitly indicated T2DM prevention in the study title or study objectives. (4) Published from 2019 to 2024. (5) Published in English. To provide a comprehensive overview of this emerging field, we included manuscripts reporting protocol descriptions and intervention development, in addition to those presenting data on feasibility, acceptability, and effectiveness. Studies involving participants of all ages were considered given the increasing prevalence of T2DM among younger populations [47].

**Fig. 1:**
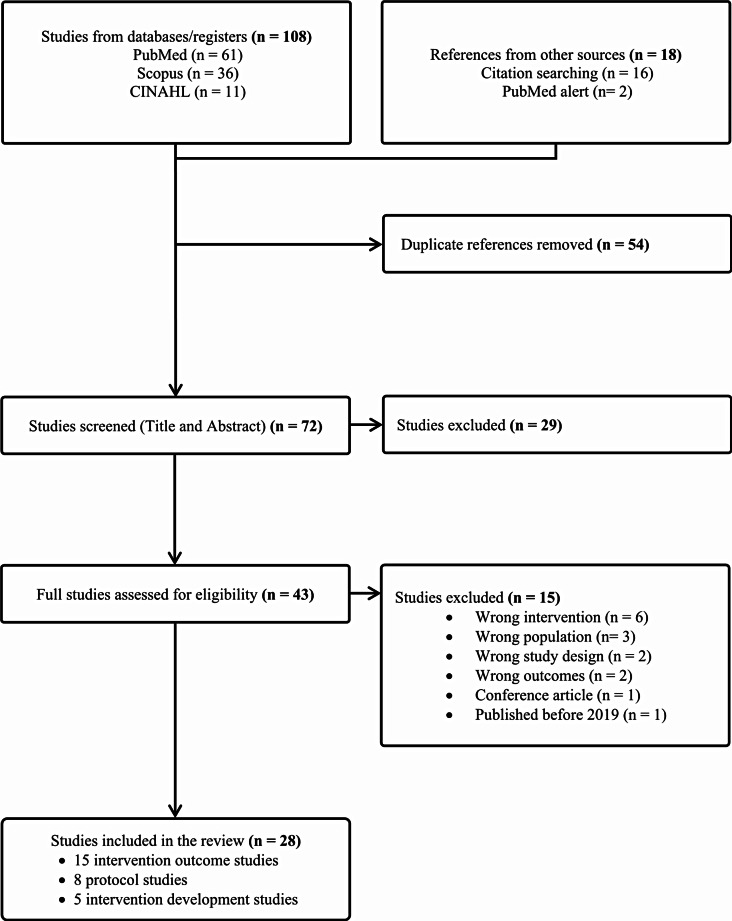
PRISMA flowchart of the study selection

We utilized the web-based software Covidence [48] to screen manuscripts and extract pertinent data, resulting in 108 records. We also reviewed the reference sections of published review manuscripts on digital T2DM prevention interventions and set up a PubMed alert for weekly notifications of newly published manuscripts. After removing duplicates, we screened the titles and abstracts of 72 articles. A total of 43 full-text studies were assessed for eligibility, with 28 articles meeting our inclusion criteria. A PRISMA flowchart details the study selection process (Fig. 1).

### Data Extraction

Two authors (TF and JD) independently extracted data from the articles included and reviewed each other’s extractions fully to ensure accuracy. Discrepancies were resolved through collaborative discussions during a review meeting until a consensus was reached. Data were summarized in Excel and synthesized narratively. The final database of extracted studies is available in the supplementary materials. The extracted data includes lead author, year of publication, journal, study aims, study design, year of data collection, study population, participant demographics, country/region, recruitment methods, intervention development process, T2DM prevention content, specific characteristics of the text-messaging program (e.g., frequency of messages, whether the messages were automated or tailored), outcomes, cost, and potential implications for health equity.

## Results

### Studies Overview

Out of the 28 manuscripts included, five focused on intervention development, eight described intervention protocols, and 15 were intervention outcome studies. Among the latter outcome studies, experimental designs varied: six were randomized controlled trials (RCTs) [49–54], six were single-group, pre-post studies [55–60], two were pilot RCTs [61, 62], and one was a non-randomized pragmatic effectiveness trial [63]. All protocol manuscripts detailed RCTs [64–71]. Out of the five intervention development manuscripts, four qualitatively described the phases of text-messaging development [72–75], and one included a survey where participants evaluated and refined the intervention’s text-message library [76]. Geographically, the studies were widely dispersed: 16 were conducted in the U.S., three each in Australia and the UK, and two in South Africa. Additional countries represented included India, Spain, Saudi Arabia, Sri Lanka, Bangladesh, and Thailand.

Text-messaging integration took two forms. Often, the integration of text-messaging aimed to evaluate whether additional content delivery through text-messages could enhance participant engagement, improve outcomes, sustain behavior changes, or nudge participants to enroll in in-person programs. Yet, many studies utilized text-messaging as the primary delivery method for T2DM prevention content [49, 50, 53, 56, 57, 59, 62, 68, 71, 73, 75].

### Settings and Recruitment

Many studies were integrated within healthcare settings, including primary care clinics, federally qualified health centers, and hospitals. Four studies explicitly focused on implementing text-messaging interventions within these settings, aligning with existing workflows, and using Electronic Health Record (EHR) data for recruitment [55, 58, 63, 64]. Ritchie et al. (2020) and Fischer et al. (2019) applied the SMS4PreDM program in a safety net healthcare system [58, 63]. The myAgileLife DPP study was also implemented in a large healthcare system and identified participants through laboratory data, EHR mentions of prediabetes, or direct provider referrals, with self-enrollment via a web portal to facilitate recruitment [55]. The BEGIN study conducted a pragmatic trial in a federally qualified health center, using an EHR algorithm for participant identification and an opt-out recruitment letter to explain study objectives and participation [64]. Studies also utilized community-based strategies for participant recruitment, including participation in health fairs, local events, health promotion activities, and sporting events. Additionally, studies employed various additional recruitment methods, such as research databases, invitation letters, newspaper and social media advertisements, phone calls, and emails.

### Intervention Development and Content

Studies focused on intervention development provided the most detailed insights into this process, but summaries were also available in protocols and outcome studies, though with varying levels of detail. Researchers typically employed multi-phase processes to develop text-message content. Some investigators developed the content themselves, leveraging their expertise and insights from literature reviews [51, 52, 60, 61]. Others collaborated with experts such as clinicians (physicians, dietitians), certified lifestyle change educators/coaches, and physical activity trainers [49, 55, 56, 62, 64–66]. These collaborations often involved multiple meetings with the expert team, utilizing interviews, focus groups, or workshops. Additionally, some researchers included potential participants and individuals with prediabetes or T2DM in the program development process [50, 53, 58, 63, 67, 70, 75]. One study utilized artificial intelligence to develop the intervention content [59]. While some authors independently developed the message library and later sought feedback for refinement, others employed a fully co-designed approach, engaging collaborators throughout the development process. For instance, Hill et al. (2023) used an 11-step approach, which included evidence review, needs assessment, expert input, content development, readability and acceptability evaluation, and refinement based on participant feedback [74].

Commonly, included studies modified evidence-based content and curricula developed through RCTs, such as the DPP trial, whose curriculum includes strategies for lifestyle modification with information about nutrition, physical activity, and behavioral self-management [77]. In studies conducted in the U.S., authors often utilized the National DPP curriculum, which is based on the DPP trial materials [78]. Adaptations to the curriculum were sometimes made to suit the population served or the context in which the study was being tested. For studies outside the U.S., adaptations of DPP content were tailored to fit the specific cultural and contextual needs of the respective countries. For example, in Lifestyle Africa the investigators adapted the National DPP curriculum to make cultural, educational, and language adaptations relevant to the local community [67]. A few studies relied on general lifestyle change content about diet and physical activity, without specific references to using content from DPP or other evidence-based programs.

As expected, the text-messages were primarily focused on promoting physical activity and healthy dietary practices for weight loss, with content and behavioral strategies around these topics similar to those included in the DPP curriculum. This included goal setting, promotion of self-efficacy, motivation, coping with barriers, stress reduction, and skill teaching (e.g., tracking calories and exercise), among others. Additionally, some studies included content about T2DM pharmacotherapies (i.e., metformin). A few studies incorporated other topics to fit the study objectives, such as sleep goals, prompts to join in-person programs, and breastfeeding.

### Text-messaging Details

#### Frequency

The frequency of messages and the duration of text-messaging programs varied widely across studies. Due to differences in program lengths and intensities, it is challenging to estimate the average number of messages typically sent as part of the interventions. Some programs were more intensive, sending multiple messages per day (up to four), while others sent a few messages per week or only monthly messages (Table 1).

**Table 1:** Characteristics of studies on text-messaging interventions for type 2 diabetes prevention

Author (Year)	Intervention duration	Intensity	Message type	Digital delivery method
**Intervention outcome studies**				
Brown S (2024) [54]	5 months (24-month follow-up)	10 messages (total)	Standardized automated unidirectional	Text + phone calls
Cheung N (2024) ^a^ [49]	6 months	4/week	Standardized automated unidirectional + tailored	Text + wearable fitness tracker
Arora S (2023) [55]	12 months	Up to 3/day	Standardized automated unidirectional + tailored + bidirectional automated on-demand + live interactions	Text + webpage
Formagini T (2023) [56]	6 months	Up to 3/day	Standardized automated unidirectional + bidirectional automated on-demand + live interactions	Text only
Stewart J (2022) [57]	4 months (6-month follow-up)	Daily (number not provided)	Standardized automated unidirectional + tailored	Text + wearable fitness tracker
Bootwong P (2022) [50]	8 weeks (12-week follow-up)	5/week	Standardized automated unidirectional	Text only
Khunti K (2021) [51]	12 months (48-month follow-up)	1/week in months 1–6; 1/month in months 7–12	Standardized automated unidirectional + tailored	Text + phone calls
Staite E (2020) [52]	12 months	3–4/day	Standardized automated unidirectional	Text + wearable fitness tracker + mobile app + web-based sessions
Rollo M (2020) [61]	3 months	8 messages (total)	Standardized automated unidirectional + tailored	Text + webpage + telehealth coaching via video call
Nanditha A (2020) [53]	24 months	2–3/week	Standardized automated unidirectional + tailored	Text only
Ritchie N (2020) ^b^ [63]	12 months	6 days per week for 12 months	Standardized automated unidirectional	Text only
Fischer H (2019) ^b^ [58]	12 months	6 days per week for 12 months	Standardized automated unidirectional	Text only
Cheung N (2019) ^a^ [62]	26 weeks (6-month follow-up)	Up to 3/week	Standardized automated unidirectional + tailored	Text + wearable fitness tracker + phone call
Stephens T (2019) [59]	Not described	4,123 total	Automated bidirectional tailored (AI)	Text only
Kim M (2019) [60]	6 months	120 total	Standardized automated unidirectional	Text + online training modules
**Intervention protocol studies**				
Vargas M (2023) [64]	12 months	2/week in months 1–6, and 1/week in months 7–12	Standardized automated unidirectional + bidirectional automated on-demand	Text only
Soltero E (2023) ^c^ [71]	12 weeks	2–3/day	Standardized automated unidirectional + bidirectional automated on-demand	Text + wearable fitness tracker
Carter E (2023) [65]	12 months (24-month follow-up)	5/week + 3/month	Standardized automated unidirectional + bidirectional automated on-demand	Text only
Galmes-Panades A (2022) [66]	6 months	3–5/week	Standardized automated unidirectional + tailored	Text + online session
Catley D (2019) [67]	12 months (24-month follow-up)	2/day	Standardized automated unidirectional	Text + weekly in-person sessions with community health workers
Alzeidan R (2019) [68]	6 months (36-month follow-up)	3/week	Standardized automated unidirectional + tailored	Text only
Gupta Y (2019) [69]	12 months (24-month follow-up)	2/week	Standardized automated unidirectional	Text + phone calls
Sinclair K (2020) [70]	12 months	2/week	Standardized automated unidirectional	Text only
**Intervention development studies**				
Soltero E (2023) ^c^ [75]	Not described	116 total	Standardized automated unidirectional + tailored	Not described
Hill J (2023) [74]	Not described	67 total	Standardized automated unidirectional	Not described
MacPherson M (2021) ^d^ [76]	Not described	120 total	Standardized automated unidirectional	Not described
MacPherson M (2021) ^d^ [72]	Not described	124 total	Standardized automated unidirectional	Not described
Rodriguez D (2021) [73]	Not described	Not described	Standardized automated unidirectional + tailored	Not described

Note: See the online supplementary material for additional information about the studies included. a, b, c, d = manuscripts reporting on the same intervention

#### Tailoring

Some studies tailored messages to participants based on various characteristics. The simplest method for tailoring involved using participants’ names or sending messages at their preferred times. More sophisticated tailoring was also utilized. For example, Rollo et al. (2020) personalized messages based on barriers identified by participants as impacting their ability to eat healthfully and be physically active [61]. Nanditha et al. (2020) tailored messages according to participants’ stages of change based on the Transtheoretical Model (TTM), with messages specific to each stage (pre-contemplation, contemplation, preparation, action, and maintenance) [53]. Cheung et al. (2019) used prespecified algorithms to send personalized messages based on baseline data, such as ethnicity and breastfeeding status [62].

#### Unidirectional vs. Bidirectional Messages

While most studies allowed only one-way communication, some offered bidirectional interaction. This included automated responses based on participant replies (i.e., on-demand) or live interactions with human operators (e.g., lifestyle change coaches). Automated systems used keywords so that participants could receive follow-up messages. Some studies asked participants to reply to the messages with their weekly weight or other specific requests. In a few cases, participants could send free text-messages monitored by investigators or lifestyle coaches, who would then respond to the participants, providing support on the topics mentioned in their messages [15, 55]. In myAgileLife DPP, a lifestyle coach contacted participants at least once per module, and more often if necessary, to discuss module content and lifestyle recommendations [55].

#### Wearables

Some studies also incorporated wearable monitors, such as the Fitbit smartwatch and physical activity tracker to complement the program [49, 52, 54, 57, 62, 71]. These devices captured physical activity in real-time and provided immediate feedback through text-messages. Some also combined the Fitbit watch with the app for tracking dietary practices. For example, Soltero et al. (2023) tailored text-messages based on participants’ step counts, with weekly goals increasing incrementally [71]. In Steward et al. (2022), participants used the Fitbit app to log food consumption and physical activity, receiving personalized text content based on their entries [57].

### Participants

The smallest study, a pre-post feasibility study, included 23 participants [59], while the largest, an RCT, had 1,031 participants [53]. Consistent with the DPP translational literature, which has demonstrated the underrepresentation of men in T2DM prevention interventions [14], we observed that men were less likely to participate in the interventions included in this review. One exception was a protocol by Sinclair et al. (2020), which was specifically designed for American Indian and Alaska Native men [70].

Participants were typically older than 40 or 50 years, reflecting the higher prevalence of prediabetes in older populations [13]. Studies targeting women with a history of gestational diabetes enrolled younger participants, averaging in their thirties [49, 61, 62, 69]. Two interventions focused on children and adolescents, addressing the high prevalence of obesity in youth and their associated risk for developing T2DM [59, 71, 75]. Studies conducted in the U.S. tended to focus on racial and ethnic minority groups, such as Hispanic/Latinos, Blacks, and American Indian/Alaska Native men, or included a representative percentage of participants from diverse races and ethnicities.

T2DM risk was primarily assessed using hemoglobin A1c or fasting plasma glucose. Intervention development studies tended to be vague about inclusion criteria, generally stating participants had prediabetes or were at high risk of T2DM [72–74, 76]. For studies targeting women with previous gestational diabetes, a prior diagnosis was used to assess T2DM risk [49, 61, 62]. Notably, a few studies assessed T2DM risk solely based on overweight/obesity levels (through body mass index [BMI]) [67, 70, 71, 75], with one study justifying this approach to be more inclusive of potential participants [67].

### Feasibility and Acceptability

Table 2 presents an overview of the studies’ feasibility, acceptability, and effectiveness. Nine studies that reported outcome data included data on feasibility and/or acceptability. Feasibility was assessed through recruitment and retention rates, engagement levels (among those with bidirectional interactions), and technical issues. Acceptability was measured via surveys or qualitative interviews, focusing on participant satisfaction, perceived helpfulness, likelihood of recommending the program, and suggestions for improvement.

**Table 2:** Summary of outcomes from text-messaging interventions for type 2 diabetes prevention (2019–2024)

Author (Year)	Feasibility	Acceptability	Effectiveness
Brown S (2024) [54]	Not reported	Not reported	No statistically significant group differences were found in fasting glucose, A1c, BMI, and waist-to-hip ratio. The text-messages’ group had a lower diabetes conversion rate (22.2%) compared to the control group (28%) but a higher conversion than the in-person group (16.7%).
Cheung N (2024) ^a^ [49]	Not reported	Most women reported positive feedback about the text messaging program, and that it motivated them to make healthier choices.	A total of 12% of the intervention group and 11% of the control group achieved the Healthy Lifestyle Outcome (a composite score of weight, physical activity and diet) at six months (RR 1.15, 95% Cl 0.41–3.20, *p* = 0.79).
Arora S (2023) [55]	The mean program duration was 265 days (SD = 125). On average, participants attended 12 sessions (SD = 8). Program engagement rates included 82% of sessions with text-messages (SD = 24), 61% with logged activity minutes (SD = 35), and 72% with logged weight (SD = 25). Of the 163 participants, 54% completed the 12-month program.	Not reported	Mean weight loss was 5.5% at six months and 4.3% at 12 months (*p* < 0.001). HbA1c decreased from 6.1 to 5.8 at both six and 12 months (*p* < 0.001), with no significant increase in physical activity.
Formagini T (2023) [56]	Participants sent an average of 38.9 messages (SD = 25.6), mostly free text (mean = 36.5, SD = 24.6), ranging from 3 to 147. Over half of participants (58.3%) reported technical issues, and 92% (24 of 26) completed the program and follow-up.	All participants were satisfied with the program (83.3% very, 16.7% extremely). While 66.7% were uncertain about responses to their free-text messages, 87.5% of participants felt that the program helped prevent diabetes.	Mean body weight dropped from 191.2 to 186.7 pounds (*p* = 0.004); 45.8% lost ≥ 3%, and 29.2% lost ≥ 5%. Waist circumference decreased by 1.1 cm (*p* = 0.03). Physical activity frequency increased (*p* = 0.003). Diet quality and A1c did not change. Five participants (20.8%) reversed to normoglycemia.
Stewart J (2022) [57]	Not reported	Not reported	Participants experienced significant reductions in weight at six months (-3.3 kg, *p* = 0.026) and BMI (-1.25 kg/m², *p* = 0.005). Physical activity increased, with 2 more moderate activity days per week (95% Cl: 0.4 to 3.6; *p* = 0.015), 1.5 more vigorous activity days per week (95% Cl: 0.1 to 2.9; *p* = 0.035), and 62 min of activity per week (*p* = 0.039). Sedentary time decreased from 509.5 to 388 min per day (*p* = 0.007).
Bootwong P (2022) [50]	Not reported	Not reported	At 8 weeks, mean physical activity increased significantly in the intervention group (+ 1,590.73 METs/min/week) vs. control (+ 407.39 METs/min/week), adjusted difference: -1,183 METs/min/week (*p* = 0.02). By 12 weeks, no significant difference (adjusted difference 273.3 METs/min/week, *p* = 0.513), though moderate physical activity rose (+ 256.40 METs/min/week, *p* = 0.040). Mean waist circumference dropped by 1.21 cm in the intervention and 0.21 cm in the control group (*p* = 0.02).
Khunti K (2021) [51]	Not reported	Not reported	At 12 months, the SMS group increased ambulatory activity by 547 steps vs. control, driven by purposeful movement. Moderate-to-vigorous physical activity rose by 3.5 min/day and walking time by 8.5 min/day. These effects, including higher odds of meeting activity guidelines, were not sustained at 48 months. Small reductions in weight (1 kg) and waist circumference (1.6 cm) persisted at both 12 and 48 months.
Staite E (2020) [52]	Approximately half of those screened were eligible and consented to randomization. Of 192 participants, 80% in the intervention arms adhered to the intervention. The retention rate was 70.4%, with 69 out of 98 participants completing follow-up.	Not reported	There was no treatment effect on weight at six months (mean difference 0.15; 95% CI − 0.93 to 1.23) or 12 months (mean difference 0.07 kg; 95% CI − 1.29 to 1.44) or for physical activity levels at six months (mean difference − 382.90 steps; 95% CI − 860.65 to 94.85) or 12 months (mean difference 92.64 steps; 95% CI − 380.92 to 566.20).
Rollo M (2020) [61]	Out of 327 potential participants, 42 were eligible and randomized. A total of 71% of participants completed the study at six months.	A total of 84% found the text-messages informative, appreciating them as gentle reminders for accountability, though some felt they were overly focused on weight. Only 22–31% agreed the messages increased confidence in improving diet or activity, and fewer (8–15%) felt they helped achieve health goals. Overall satisfaction was 39%, with suggestions for more motivational content.	No significant group-by-time effects were observed for most outcomes. For body weight, a trend favoring the intervention groups was observed at three months and six months, although the differences among the three groups were not significant (*p* = 0.29).
Nanditha A (2020) [53]	Not reported	Not reported	Over the 2-year follow-up, 22.7% of the control group and 21.0% of the intervention group developed diabetes, with no significant effect of the intervention on diabetes progression. Mean values of outcome measures showed minimal change from baseline in both groups.
Ritchie N (2020) ^b^ [63]	Not reported	Not reported	Mean values of most outcomes changed little between baseline and any of the follow-up visits in either randomized group.
Fischer H (2019) ^b^ [58]	SMS4PreDM participants had high retention (259 of 285 patients or 91.0% completion at 12-months.	Not reported	SMS4PreDM participants had a time-related mean weight loss of 1.3 pounds (SE 0.74). Overall, frequency of achieving ≥ 3% weight loss was comparable between groups.
Cheung N (2019) ^a^ [62]	Participants sent 228 text-messages: 98 were simple responses (e.g., “thank you”), 85 related to activity monitor issues (e.g., malfunctioning), 27 about logistics, and 18 on other topics. Activity monitor data showed that 6 participants never wore the Fitbit, 27 experienced at least one issue with it, 4 lost their Fitbit, and on 18 occasions, it was reported lost or damaged. A total of 85% found the Fitbit useful and checked their results most days.	The vast majority found the SMS helpful, although the reported effects on diet and physical activity were modest.	There was no significant difference in postpartum glucose tolerance test completion rates between the control (65%) and intervention (70%) groups. One participant in each group developed diabetes, and glucose levels were similar (fasting glucose: 4.9 ± 0.7 vs. 5.0 ± 4.2 mmol/L, *p* = 0.9; 2-hour glucose: 7.2 ± 2.2 vs. 6.4 ± 2.0 mmol/L, *p* = 0.2). Dietary and physical activity targets were not significantly different between groups.
Stephens T (2019) [59]	A total of 4,123 messages were exchanged with the program, resulting in 269 conversations (average 12 per patient, SD = ± 8.84). The longest conversation lasted 1 h and 13 min, while the shortest ranged from 4 to 7 s	Adolescent patients reported positive progress toward their goals 81% of the time. Patients’ reported usefulness ratings 96% of the time, which illustrate that adolescents engaged with and viewed this chatbot as helpful.	Not reported
Kim M (2019) [60]	During the six-month recruitment period, 311 participants were recruited, 247 met the eligibility criteria and enrolled in the program, and 215 completed the intervention and follow-up outcome evaluation.	The intervention was well accepted, with older adults adapting to the technology-assisted components due to the bilingual Korean CHWs, a Korean-translated interface, and interactive texting in Korean, finding the digital approach acceptable with added human support.	The group with prediabetes reduced A1C by − 0.4% at 3 months and − 0.6% at 6 months, and slightly less than two thirds (63.6%) of this group successfully lowered A1C below 5.7%. No statistically significant changes from baseline to six months was found among the group with prediabetes (*p* = 0.362).

Note: See the online supplementary material for additional information about the studies included. a, b = manuscripts reporting on the same intervention

Retention rates were consistently high across studies, with more than 80% completion reported in seven studies [50, 52, 53, 56, 58, 60, 61]. Among the studies that utilized bidirectional interactions, engagement did not seem to be an issue [55, 56, 59]. More than 4,000 messages were exchanged among the 23 teenage participants in the Tess study, which used an AI behavioral coaching chatbot [59]. Participants in the myAgileLife DPP sent text-messages during 82% of the modules, logged activity minutes in 61%, and recorded weight in 72% [55]. Despite these positive outcomes, technical issues were occasionally reported [56, 62].

The acceptability findings showed variability across studies. Overall, participants rated the studies with high levels of satisfaction and positive feedback, but perceptions of helpfulness varied, and suggestions for improvement were noted (see Table 2). In one study, most participants reported high satisfaction with the program, but many (66%) indicated uncertainty about their opinions on the responses they got from their texts [56]. Another study revealed that while 84% of women found the messages useful, they also highlighted concerns regarding the emphasis on weight outcomes [61]. Additionally, preferences for in-person interactions over digital communication were noted in this study [61]. Similarly, a study involving older adults reported high acceptance of technology, provided it was accompanied by human interactions with support from community health workers [60].

### Effectiveness

Consistent with the T2DM prevention literature, studies commonly assessed changes in physical activity, dietary behaviors, and weight loss. Physical activity was evaluated through self-reported and objective measures, while dietary behaviors were only assessed via self-report. Body weight reduction, typically measured as a percentage of baseline weight, was the most frequent biomarker of programs’ effectiveness. Some studies also reported glycemic markers such as hemoglobin A1c, fasting plasma glucose levels, or progression to T2DM. Secondary biomarkers often included were BMI, waist circumference, waist-to-hip ratio, blood pressure, and cholesterol levels. Although less common, some studies examined sedentary time, quality of life, sleep duration, self-efficacy, depressive symptoms, and alcohol intake.

The effectiveness of the interventions varied widely, reflecting the field’s ongoing development. Of the 12 studies using weight loss as an outcome, only five reported statistically significant reductions or trends in weight post-intervention [55–57, 60, 61]. Glycemic markers were measured in eight studies, with two showing significant reductions [55, 60]. Although two studies did not find statistically significant reductions, one noted that 21% of participants reverted to normal glycemic levels post-intervention [56]. Another study reported a lower T2DM conversion rate (22.2%) in the text-messaging group compared to the control (28%) [54]. Similarly, some studies reported increases in physical activity and improved dietary behaviors [50, 51, 56, 57], though results were inconsistent.

Drawing definitive conclusions is challenging due to the limited number of studies and even fewer fully powered RCTs. However, a few trends emerged. First, interventions with higher participant interaction generally showed more favorable outcomes. Second, programs with daily messages and additional features tended to be more successful than low-touch interventions that relied solely on infrequent automated messages. For instance, a 12-week intervention consisting of eight text-messages showed no improvements in body weight, physical activity, or A1c levels [61]. In contrast, a highly interactive program with three messages per day, including interactive and on-demand messages, plus web-based resources and live coaching, resulted in a mean weight loss of 5.5% at six months and 4.3% at twelve months (*p* < 0.001) [55]. Another study found that 46% of participants lost 3% of body weight and 29% lost 5% post-intervention, with 2–3 daily automated messages, on-demand messages, and live coaching [56].

### Cost

Detailed cost information was available in three manuscripts. The SMS4PreDM intervention, which supplemented an in-person DPP with six daily messages for 12 months, cost approximately $100.92 per participant [58, 63]. The DPPFit study, which adapted the National DPP curriculum with daily text messages for 16 weeks, cost $2.26 per participant [57]. Although comprehensive cost data were sparse across most studies, text-messaging interventions appear considerably less costly than traditional in-person DPP programs. For instance, a 2016 report from a large urban health system cited a cost of $553 per participant for in-person DPP [18], highlighting the potential cost savings of SMS4PreDM, DPPFit, and similar interventions.

## Discussion

This review evaluated recent literature on text-messaging interventions for T2DM prevention published between 2019 and 2024. While text-messaging is a widely used approach for delivering behavior change interventions [36–40], its application in T2DM prevention remains an evolving area of research. Notably, many studies included in our review are early-stage research, including intervention development studies, protocols, and pilot trials. This pattern underscores the field’s current developmental phase and suggests that findings from these preliminary studies may lay the groundwork for more definitive evidence in the next 3–5 years. The following sections discuss critical gaps, future research directions, and opportunities to promote health equity in T2DM prevention.

### Gaps and Future Directions

A notable gap in the reviewed literature is the predominant reliance on one-way messaging systems with low intensity and limited participant engagement, which were often associated with minimal behavioral or biomarker improvements. The limited number of studies included in our review that employed higher levels of participant interaction and more intensive contact generally demonstrated more favorable outcomes. Based on these results, future interventions could explore prioritizing the development of more interactive and engaging text-messaging content. Similarly, the effectiveness of text-messaging programs may be enhanced by integrating features such as personalized coaching messages, interactive response mechanisms, and the utilization of wearable technologies for real-time activity tracking. These enhancements may be critical for improving retention and engagement, particularly considering that digital interventions often forfeit the human interactions inherent in in-person interventions. Thus, innovative strategies will be essential for maintaining participant engagement throughout the intervention process.

Another notable gap was the limited integration of social support mechanisms within the interventions, despite their well-documented benefits for healthy behavior change and the importance of social support in DPP [79–82]. Only a few studies in our review included interactions with a lifestyle change coach. In-person DPP sessions are often delivered in a group format, facilitating accountability and social support. Because text-messaging is inherently a more individualized strategy, this structure may limit opportunities for social connection, highlighting the need for innovative approaches to embedding social support within these interventions. For example, involving friends and family members might foster collective behavior change and mutual support within households. Digital platforms such as WhatsApp or Facebook groups could facilitate virtual communities where participants can interact, share progress, and support each other, mimicking successful social support structures seen in traditional in-person DPP settings.

Methodological limitations also need to be addressed in future work. For example, most studies used self-reports of physical activity or did not include dietary assessments. While understandable for logistical reasons, this approach could introduce bias in measuring intervention impacts related to healthy lifestyle changes. Future research should prioritize incorporating objective measures where feasible, such as wearable devices for activity tracking and validated dietary assessment tools. Enhancing the precision of dietary assessment is critical for understanding the impact of interventions on diet quality, a key determinant of T2DM risk reduction. Addressing these gaps will enable more robust evaluations of text-messaging interventions’ effectiveness in improving T2DM-related health outcomes.

### Health Equity Considerations

The role of text-messaging interventions in potentially achieving health equity in T2DM prevention reveals promising progress and key considerations for future research. As discussed, many studies specifically targeted racial and ethnic minority groups or included an inclusive sample of participants from diverse races and ethnicities. Given the higher prevalence of T2DM in racial and ethnic minorities [13], this targeted focus suggests an active effort by investigators to address these T2DM-related disparities. Yet, most studies were conducted in the U.S., highlighting the need for similar efforts in low- and middle-income countries, where insights into adaptability across diverse healthcare settings are essential.

Additionally, tailoring interventions to meet the cultural, educational, and linguistic needs of participants was a common approach, often achieved through collaborations with safety-net organizations and community health centers that often serve uninsured or underinsured individuals. Tailoring programs and developing these partnerships for participant recruitment underscore the potential of text-messaging interventions to improve access to T2DM prevention for those who traditionally do not benefit from in-person programs [83, 84]. Furthermore, community and stakeholder involvement in developing these interventions, frequently reported across the included studies, is recognized as a crucial component for successful implementation and sustainability [85, 86].

An additional observation is the increased reliance on smartphones as text-messaging interventions become more interactive and dynamic. Enhanced capabilities—such as embedded links, multimedia content, and interactive elements—support more engaging and innovative program experiences, moving beyond simple, text-only messages. However, reliance on smartphones for such content may limit the reach of individuals without access to this technology. Although smartphone access is widespread in countries like the U.S., this reliance could present barriers in resource-limited settings where smartphone availability is less universal. While providing smartphones to participants could help address this gap, practical and financial feasibility remains a challenge.

A final consideration is the limited discussion in the included studies regarding how investigators supported participants with low literacy or technological proficiency. The digital divide, often discussed in the context of digital interventions, refers to the disparities in access to technology and digital literacy skills that can affect individuals’ ability to engage with health programs [87]. One potential issue is that participants with lower literacy levels may disengage or drop out due to challenges in interacting with the program content. Actively supporting these individuals—through tailored training, simplified content, and ongoing assistance—could enhance engagement and efficacy, ultimately contributing to more equitable outcomes in T2DM prevention.

### Limitations

This review has notable limitations. Although we employed a systematic approach for study selection, some relevant studies may have been unintentionally excluded, possibly limiting the scope of our findings. Additionally, because the sample size was relatively small (*n* = 28) and some studies lacked outcome data, our conclusions are restricted to the studies included. We did not conduct a formal quality assessment of each study, which may have hindered our ability to identify potential biases and variability in study rigor, thereby affecting the reliability of our findings. Finally, as this review did not aim to synthesize quantitative findings through meta-analysis, conclusions regarding the effectiveness of interventions should be interpreted with caution.

## Conclusion

Text-messaging interventions hold promise as scalable tools for T2DM prevention, leveraging their potential to reach diverse populations across varied settings. This review underscores their feasibility and acceptability, although their effectiveness remains mixed. Challenges persist in optimizing engagement, ensuring the social support crucial to DPP is maintained, and conducting rigorous, large-scale pragmatic trials to establish their effectiveness and sustainability. The potential for scalability of text-messaging programs for T2DM prevention will also require rigorous estimation of cost-effectiveness, which is needed for many stakeholders to adopt such programs. Addressing these areas will be pivotal in advancing text-messaging interventions from promising innovations to impactful solutions in T2DM prevention.

## Key References

- Nguyen V, Ara P, Simmons D, Osuagwu UL. The Role of Digital Health Technology Interventions in the Prevention of Type 2 Diabetes Mellitus: A Systematic Review. Clinical Medicine Insights: Endocrinology and Diabetes. 2024;17:11795514241246419.
-
  - This review shows preliminary evidence of the effectiveness of digital health technologies in improving diabetes risk-related outcomes.
- Gentili A, Failla G, Melnyk A, Puleo V, Tanna GLD, Ricciardi W, et al. The cost-effectiveness of digital health interventions: a systematic review of the literature. Frontiers in Public Health. 2022;10:787135.
-
  - Findings from this review indicate a growing body of evidence supporting the generally favorable effects of digital health interventions on costs and health outcomes.
- MacPherson MM, Merry KJ, Locke SR, Jung ME. mHealth prompts within diabetes prevention programs: A scoping review. Mhealth. 2022;8.
-
  - This scoping review found mixed evidence on the effectiveness of text-messaging and other digital technology prompts in behavioral outcomes and diabetes incidence up to 2020.

## Electronic Supplementary Material

Below is the link to the electronic supplementary material.

Supplementary Material 1

## Data Availability

Data is provided within the supplementary information files.

## References

[1] Knowler WCB-CE, Fowler SE, Hamman RF, Lachin JM, Walker EA, Nathan DM, Diabetes Prevention Program Research Group. Reduction in the incidence of type 2 diabetes with lifestyle intervention or metformin. N Engl J Med. 2002;346(6):393–403. 11832527 10.1056/NEJMoa012512 PMC1370926

[2] Pan X-R, Li G-w, Hu Y-H, Wang J-X, Yang W-Y, An Z-X, et al. Effects of diet and exercise in preventing NIDDM in people with impaired glucose tolerance: the Da Qing IGT and Diabetes Study. Diabetes Care. 1997;20(4):537–44. 9096977 10.2337/diacare.20.4.537

[3] Ramachandran A, Snehalatha C, Mary S, Mukesh B, Bhaskar A, Vijay V, et al. The Indian Diabetes Prevention Programme shows that lifestyle modification and metformin prevent type 2 diabetes in Asian Indian subjects with impaired glucose tolerance (IDPP-1). Diabetologia. 2006;49:289–97. 16391903 10.1007/s00125-005-0097-z

[4] Lindstrom J, Louheranta A, Mannelin M, Rastas M, Salminen V, Eriksson J, et al. The Finnish diabetes Prevention Study (DPS) Lifestyle intervention and 3-year results on diet and physical activity. Diabetes Care. 2003;26(12):3230–6. 14633807 10.2337/diacare.26.12.3230

[5] Ali MK, Echouffo-Tcheugui JB, Williamson DF. How effective were lifestyle interventions in real-world settings that were modeled on the diabetes Prevention Program? Health Aff. 2012;31(1):67–75. 10.1377/hlthaff.2011.1009 22232096

[6] Aziz Z, Absetz P, Oldroyd J, Pronk NP, Oldenburg B. A systematic review of real-world diabetes prevention programs: learnings from the last 15 years. Implement Sci. 2015;10:1–17. 26670418 10.1186/s13012-015-0354-6 PMC4681022

[7] Mudaliar U, Zabetian A, Goodman M, Echouffo-Tcheugui JB, Albright AL, Gregg EW, et al. Cardiometabolic risk factor changes observed in diabetes prevention programs in US settings: a systematic review and meta-analysis. PLoS Med. 2016;13(7):e1002095. 27459705 10.1371/journal.pmed.1002095 PMC4961455

[8] Albright AL, Gregg EW. Preventing type 2 diabetes in communities across the US: the National Diabetes Prevention Program. Am J Prev Med. 2013;44(4):S346–51. 23498297 10.1016/j.amepre.2012.12.009 PMC4539613

[9] Centers for Disease Control and Prevention (CDC). Diabetes Prevention Recognition Program Application. Registry of All Recognized Organizations: U.S. Department of Health & Human Services; [ https://dprp.cdc.gov/Registry

[10] Centers for Disease Control and Prevention (CDC). About CDC’s First National Prediabetes Awareness Campaign. U.S. Centers for Disease Control and Prevention; 2024.

[11] National DPP Coverage Toolkit. National Diabetes Prevention Program Coverage Toolkit: Participating Payers and Employers. 2020 [ https://www.ncbi.nlm.nih.gov/pmc/articles/PMC8415384/

[12] Hill-Briggs F, Adler NE, Berkowitz SA, Chin MH, Gary-Webb TL, Navas-Acien A, et al. Social determinants of health and diabetes: a scientific review. Diabetes Care. 2021;44(1):258. 10.2337/dci20-0053 PMC7783927 33139407

[13] Centers for Disease Control and Prevention (CDC). National Diabetes statistics Report. U.S. Centers for Disease Control and Prevention; 2024.

[14] Ely EK, Ng BP, Cannon MJ. Delivering the National Diabetes Prevention Program: Assessment of outcomes in In-Person and virtual organizations. J Diabetes Res. 2023;2023(1):8894593. 37928892 10.1155/2023/8894593 PMC10622599

[15] Formagini T, Brooks JV, Roberts A, Bullard KM, Zhang Y, Saelee R, et al. Prediabetes prevalence and awareness by race, ethnicity, and educational attainment among US adults. Front Public Health. 2023;11:1277657. 38164446 10.3389/fpubh.2023.1277657 PMC10758124

[16] Gruss SM, Nhim K, Gregg E, Bell M, Luman E, Albright A. Public health approaches to type 2 diabetes prevention: the US National Diabetes Prevention Program and beyond. Curr Diab Rep. 2019;19:1–11. 31385061 10.1007/s11892-019-1200-z PMC6682852

[17] Cannon MJ, Masalovich S, Ng BP, Soler RE, Jabrah R, Ely EK, et al. Retention among participants in the National Diabetes Prevention Program lifestyle change program, 2012–2017. Diabetes Care. 2020;43(9):2042–9. 32616617 10.2337/dc19-2366 PMC11000538

[18] Parsons AS, Raman V, Starr B, Zezza M, Rehm CD. Medicare underpayment for diabetes Prevention Program: implications for DPP suppliers. Am J Manag Care. 2018;24(10):475–8. 30325189

[19] Ritchie ND, Baucom KJ, Sauder KA. Current perspectives on the impact of the National Diabetes Prevention Program: building on successes and overcoming challenges. Diabetes, Metabolic Syndrome and Obesity. 2020:2949-57. 10.2147/DMSO.S218334 PMC7445538 32903871

[20] Bergman M, Buysschaert M, Schwarz PE, Albright A, Narayan KV, Yach D. Diabetes prevention: global health policy and perspectives from the ground. Diabetes Manage (London England). 2012;2(4):309. 10.2217/dmt.12.34 PMC4556601 26339296

[21] Mohan V. National Diabetes prevention programmes in LMICs are now a necessity. Lancet Global Health. 2023;11(10):e1480–1. 37734780 10.1016/S2214-109X(23)00381-9

[22] Ibrahim M, Tuomilehto J, Aschner P, Beseler L, Cahn A, Eckel RH, et al. Global status of diabetes prevention and prospects for action: a consensus statement. Diab/Metab Res Rev. 2018;34(6):e3021. 10.1002/dmrr.3021 29757486

[23] Tuomilehto J, Uusitupa M, Gregg EW, Lindström J. Type 2 diabetes prevention programs—from proof-of-concept trials to national intervention and beyond. J Clin Med. 2023;12(5):1876. 36902668 10.3390/jcm12051876 PMC10003211

[24] Cannon MJ, Ng BP, Lloyd K, Reynolds J, Ely EK. Delivering the National Diabetes Prevention Program: Assessment of Enrollment in In-Person and Virtual Organizations. Journal of Diabetes Research. 2022;2022. 10.1155/2022/2942918 PMC8804550 35118160

[25] Pew Research Center. Mobile Fact Sheet: Pew Research Center. 2021 [ https://www.pewresearch.org/internet/fact-sheet/mobile/

[26] World Economic Forum. Charted: There are more mobile phones than people in the world: Statista; 2023 [ https://www.weforum.org/agenda/2023/04/charted-there-are-more-phones-than-people-in-the-world/#:~:text=According%20to%20the%20International%20Telecommunication,Image%3A%20Statista

[27] Joiner KL, Nam S, Whittemore R. Lifestyle interventions based on the diabetes prevention program delivered via eHealth: a systematic review and meta-analysis. Prev Med. 2017;100:194–207. 28456513 10.1016/j.ypmed.2017.04.033 PMC5699208

[28] Bian RR, Piatt GA, Sen A, Plegue MA, De Michele ML, Hafez D, et al. The effect of technology-mediated diabetes prevention interventions on weight: a meta-analysis. J Med Internet Res. 2017;19(3):e76. 28347972 10.2196/jmir.4709 PMC5387112

[29] Grock S, Ku J-h, Kim J, Moin T. A review of technology-assisted interventions for diabetes prevention. Curr Diab Rep. 2017;17:1–12. 28942537 10.1007/s11892-017-0948-2

[30] Nguyen V, Ara P, Simmons D, Osuagwu UL. The role of Digital Health Technology Interventions in the Prevention of type 2 diabetes Mellitus: a systematic review. Clin Med Insights: Endocrinol Diabetes. 2024;17:11795514241246419. 38779330 10.1177/11795514241246419 PMC11110501

[31] Villegas V, Shah A, Manson JE, Tobias DK. Prevention of type 2 diabetes through remotely administered lifestyle programs: a systematic review. Contemp Clin Trials. 2022;119:106817. 35691488 10.1016/j.cct.2022.106817

[32] Van Rhoon L, Byrne M, Morrissey E, Murphy J, McSharry J. A systematic review of the behaviour change techniques and digital features in technology-driven type 2 diabetes prevention interventions. Digit Health. 2020;6:2055207620914427. 32269830 10.1177/2055207620914427 PMC7093696

[33] Buss VH, Leesong S, Barr M, Varnfield M, Harris M. Primary prevention of cardiovascular disease and type 2 diabetes mellitus using mobile health technology: systematic review of the literature. J Med Internet Res. 2020;22(10):e21159. 33118936 10.2196/21159 PMC7661239

[34] Teo JYC, Ramachandran HJ, Jiang Y, Seah CWA, Lim ST, Nguyen HD, et al. The characteristics and acceptance of technology-enabled diabetes prevention programs (t‐DPP) amongst individuals with prediabetes: a scoping review. J Clin Nurs. 2023;32(17–18):5562–78. 36775886 10.1111/jocn.16649

[35] Muralidharan S, Ranjani H, Anjana RM, Allender S, Mohan V. Mobile health technology in the prevention and management of type 2 diabetes. Indian J Endocrinol Metabol. 2017;21(2):334–40. 10.4103/ijem.IJEM_407_16 PMC5367240 28459035

[36] Hall AK, Cole-Lewis H, Bernhardt JM. Mobile text messaging for health: a systematic review of reviews. Annu Rev Public Health. 2015;36:393–415. 25785892 10.1146/annurev-publhealth-031914-122855 PMC4406229

[37] Siopis G, Chey T, Allman-Farinelli M. A systematic review and meta‐analysis of interventions for weight management using text messaging. J Hum Nutr Dietetics. 2015;28:1–15. 10.1111/jhn.12207 24480032

[38] Smith DM, Duque L, Huffman JC, Healy BC, Celano CM. Text message interventions for physical activity: a systematic review and meta-analysis. Am J Prev Med. 2020;58(1):142–51. 31759805 10.1016/j.amepre.2019.08.014 PMC6956854

[39] Stowell M, Dobson R, Garner K, Baig M, Nehren N, Whittaker R. Digital interventions for self-management of prediabetes: a scoping review. PLoS ONE. 2024;19(5):e0303074. 38728296 10.1371/journal.pone.0303074 PMC11086829

[40] Alamnia TT, Tesfaye W, Kelly M. The effectiveness of text message delivered interventions for weight loss in developing countries: a systematic review and meta-analysis. Obes Rev. 2022;23(1):e13339. 34519151 10.1111/obr.13339

[41] Gentili A, Failla G, Melnyk A, Puleo V, Tanna GLD, Ricciardi W, et al. The cost-effectiveness of digital health interventions: a systematic review of the literature. Front Public Health. 2022;10:787135. 36033812 10.3389/fpubh.2022.787135 PMC9403754

[42] Cartujano-Barrera F, Arana-Chicas E, Ramírez-Mantilla M, Perales J, Cox LS, Ellerbeck EF et al. Every day I think about your messages: assessing text messaging engagement among latino smokers in a mobile cessation program. Patient Prefer Adherence. 2019;22(13):1213–1219. 10.2147/PPA.S209547 PMC6659777 31413549

[43] Patton SR, Coffman MJ, De Haven MJ, Miller C, Krinner LM. Text message intervention for latino adults to improve diabetes outcomes. Hispanic Health Care Int. 2022;20(4):248–55. 10.1177/15404153221084610 35274994

[44] GlobalStats S. Desktop vs Mobile vs Tablet Market Share Worldwide. Dec 2010-May 2024 2024 [ https://gs.statcounter.com/platform-market-share/desktop-mobile-tablet/worldwide/#monthly-201012-202405

[45] Rinaldi G, Hijazi A, Haghparast-Bidgoli H. Cost and cost-effectiveness of mHealth interventions for the prevention and control of type 2 diabetes mellitus: a systematic review. Diabetes Res Clin Pract. 2020;162:108084. 32061819 10.1016/j.diabres.2020.108084

[46] MacPherson MM, Merry KJ, Locke SR, Jung ME. mHealth prompts within diabetes prevention programs: a scoping review. Mhealth. 2022;8. 10.21037/mhealth-21-22 PMC9014231 35449504

[47] Perng W, Conway R, Mayer-Davis E, Dabelea D. Youth-onset type 2 diabetes: the epidemiology of an awakening epidemic. Diabetes Care. 2023;46(3):490–9. 36812420 10.2337/dci22-0046 PMC10090267

[48] Covidence systematic review software, Melbourne. Australia: Veritas Health Innovation; [Available from: www.covidence.org.

[49] Cheung NW, Simmons D, Marschner S, Thiagalingam A, Pasupathy D, Smith BJ, et al. Randomised controlled trial of a Customised text messaging and activity monitor program for Lifestyle Improvement after Gestational Diabetes. Nutrients. 2024;16(6):820. 38542731 10.3390/nu16060820 PMC10974605

[50] Bootwong P, Intarut N. The effects of text messages for promoting physical activities in prediabetes: a randomized controlled trial. Telemedicine e-Health. 2022;28(6):896–903. 34619066 10.1089/tmj.2021.0303

[51] Khunti K, Griffin S, Brennan A, Dallosso H, Davies MJ, Eborall HC, et al. Promoting physical activity in a multi-ethnic population at high risk of diabetes: the 48-month PROPELS randomised controlled trial. BMC Med. 2021;19(1):130. 34078362 10.1186/s12916-021-01997-4 PMC8173914

[52] Staite E, Bayley A, Al-Ozairi E, Stewart K, Hopkins D, Rundle J, et al. A wearable technology delivering a web-based diabetes prevention program to people at high risk of type 2 diabetes: randomized controlled trial. JMIR mHealth uHealth. 2020;8(7):e15448. 32459651 10.2196/15448 PMC7391669

[53] Nanditha A, Thomson H, Susairaj P, Srivanichakorn W, Oliver N, Godsland IF, et al. A pragmatic and scalable strategy using mobile technology to promote sustained lifestyle changes to prevent type 2 diabetes in India and the UK: a randomised controlled trial. Diabetologia. 2020;63:486–96. 31919539 10.1007/s00125-019-05061-y PMC6997257

[54] Brown SA, Winter MA, Becker HA, García AA, Velasquez MM, Tanaka H, et al. Transitioning from an In-Person intervention to Augmented text Messaging during COVID-19 in Mexican americans with prediabetes: the Starr County Diabetes Prevention Randomized Clinical Trial. Sci Diabetes Self-management Care. 2024;50(2):107–15. 10.1177/26350106241233475 PMC11062239 38454633

[55] Arora S, Lam CN, Burner E, Menchine M. Implementation and evaluation of an Automated text message–based Diabetes Prevention Program for adults with pre-diabetes. J Diabetes Sci Technol. 2024;18(5):1139–45. 10.1177/19322968231162601 PMC11418517 36946537

[56] Formagini T, Teruel Camargo J, Perales-Puchalt J, Drees BM, Fracachan Cabrera M, Ramírez M. A culturally and linguistically adapted text-message diabetes Prevention Program for latinos: feasibility, acceptability, and preliminary effectiveness. Translational Behav Med. 2024;14(2):138–47. 10.1093/tbm/ibad053 PMC11491928 37715986

[57] Stewart JL, Hatzigeorgiou C, Davis CL, Ledford CJ. DPPFit: developing and testing a technology-based adaptation of the Diabetes Prevention Program (DPP) to address prediabetes in a primary care setting. J Am Board Family Med. 2022;35(3):548–58. 10.3122/jabfm.2022.03.210415 35641047

[58] Fischer HH, Durfee MJ, Raghunath SG, Ritchie ND. Short message service text message support for weight loss in patients with prediabetes: pragmatic trial. JMIR Diabetes. 2019;4(2):e12985. 30985289 10.2196/12985 PMC6487341

[59] Stephens TN, Joerin A, Rauws M, Werk LN. Feasibility of pediatric obesity and prediabetes treatment support through Tess, the AI behavioral coaching chatbot. Translational Behav Med. 2019;9(3):440–7. 10.1093/tbm/ibz043 31094445

[60] Kim MT, Kim KB, Nguyen TH, Ko J, Zabora J, Jacobs E, et al. Motivating people to sustain healthy lifestyles using persuasive technology: a pilot study of Korean americans with prediabetes and type 2 diabetes. Patient Educ Couns. 2019;102(4):709–17. 30391298 10.1016/j.pec.2018.10.021 PMC6440831

[61] Rollo ME, Baldwin JN, Hutchesson M, Aguiar EJ, Wynne K, Young A, et al. The feasibility and preliminary efficacy of an eHealth lifestyle program in women with recent gestational diabetes mellitus: a pilot study. Int J Environ Res Public Health. 2020;17(19):7115. 32998401 10.3390/ijerph17197115 PMC7579575

[62] Cheung NW, Blumenthal C, Smith BJ, Hogan R, Thiagalingam A, Redfern J, et al. A pilot randomised controlled trial of a text messaging intervention with customisation using linked data from wireless wearable activity monitors to improve risk factors following gestational diabetes. Nutrients. 2019;11(3):590. 30862052 10.3390/nu11030590 PMC6470941

[63] Ritchie ND, Gutiérrez-Raghunath S, Durfee MJ, Fischer H. Supplemental text message support with the National Diabetes Prevention Program: pragmatic comparative effectiveness trial. JMIR mHealth uHealth. 2020;8(6):e15478. 32554385 10.2196/15478 PMC7333069

[64] Vargas MC, Pineda GJ, Talamantes V, Toledo MJL, Owen A, Carcamo P, et al. Design and rationale of behavioral nudges for diabetes prevention (BEGIN): a pragmatic, cluster randomized trial of text messaging and a decision aid intervention for primary care patients with prediabetes. Contemp Clin Trials. 2023;130:107216. 37169219 10.1016/j.cct.2023.107216 PMC10330561

[65] Carter EW, Vadari HS, Stoll S, Rogers B, Resnicow K, Heisler M, et al. Study protocol: behavioral economics and self-determination theory to change diabetes risk (BEST change). Contemp Clin Trials. 2023;124:107038. 36460265 10.1016/j.cct.2022.107038 PMC10259647

[66] Galmes-Panades AM, Angullo E, Mira-Martínez S, Bennasar-Veny M, Zamanillo-Campos R, Gómez-Juanes R, et al. Development and evaluation of a digital health intervention to prevent type 2 diabetes in primary care: the PREDIABETEXT study protocol for a randomised clinical trial. Int J Environ Res Public Health. 2022;19(22):14706. 36429423 10.3390/ijerph192214706 PMC9690330

[67] Catley D, Puoane T, Tsolekile L, Resnicow K, Fleming K, Hurley EA, et al. Adapting the Diabetes Prevention Program for low and middle-income countries: protocol for a cluster randomised trial to evaluate ‘Lifestyle Africa’. BMJ Open. 2019;9(11):e031400. 31719084 10.1136/bmjopen-2019-031400 PMC6858109

[68] Alzeidan R, Shata Z, Hassounah MM, Baghdadi LR, Hersi A, Fayed A, et al. Effectiveness of digital health using the transtheoretical model to prevent or delay type 2 diabetes in impaired glucose tolerance patients: protocol for a randomized control trial. BMC Public Health. 2019;19:1–11. 31752774 10.1186/s12889-019-7921-8 PMC6873582

[69] Gupta Y, Kapoor D, Josyula L, Praveen D, Naheed A, Desai A, et al. A lifestyle intervention programme for the prevention of type 2 diabetes mellitus among south Asian women with gestational diabetes mellitus [LIVING study]: protocol for a randomized trial. Diabet Med. 2019;36(2):243–51. 30368898 10.1111/dme.13850

[70] Sinclair Ki, Carty C, Gonzales K, Nikolaus C, Gillespie L, Buchwald D. Strong men, strong communities: design of a randomized controlled trial of a diabetes prevention intervention for American Indian and Alaska native men. Am J Men’s Health. 2020;14(4):1557988320945457. 32757825 10.1177/1557988320945457 PMC7412907

[71] Soltero EG, Lopez C, Musaad SM, O’Connor TM, Thompson D. Fit24, a digital health intervention to reduce type 2 diabetes risk among hispanic youth: protocol for a feasibility pilot study. Contemp Clin Trials. 2023;127:107117. 36775009 10.1016/j.cct.2023.107117 PMC10065958

[72] MacPherson MM, Cranston KD, Locke SR, Bourne JE, Jung ME. Using the behavior change wheel to develop text messages to promote diet and physical activity adherence following a diabetes prevention program. Translational Behav Med. 2021;11(8):1585–95. 10.1093/tbm/ibab058 PMC8604265 34008852

[73] Rodriguez DV, Lawrence K, Luu S, Yu JL, Feldthouse DM, Gonzalez J, et al. Development of a computer-aided text message platform for user engagement with a digital diabetes Prevention Program: a case study. J Am Med Inform Assoc. 2022;29(1):155–62. 10.1093/jamia/ocab206 PMC8714274 34664647

[74] Hill J, Faber M, George C, Peer N, Mulabisano T, Mostert S, et al. The development of text messages to support people at risk of diabetes in Low-Resourced communities: the South African diabetes Prevention Programme. Nutrients. 2023;15(21):4692. 37960345 10.3390/nu15214692 PMC10647382

[75] Soltero E, Lopez C, Mihail S, Hernandez A, Musaad SM, O’Connor TM, et al. An SMS text message–based type 2 diabetes Prevention Program for hispanic adolescents with obesity: qualitative co-design process. JMIR Formative Res. 2023;7(1):e46606. 10.2196/46606 PMC10433019 37531191

[76] MacPherson M, Cranston K, Johnston C, Locke S, Jung ME. Evaluation and refinement of a bank of SMS text messages to promote behavior change adherence following a diabetes prevention program: survey study. JMIR Formative Res. 2021;5(8):e28163. 10.2196/28163 PMC8433931 34448713

[77] Diabetes Prevention Program Research Group. The Diabetes Prevention Program (DPP) description of lifestyle intervention. Diabetes Care. 2002;25(12):2165–71. 12453955 10.2337/diacare.25.12.2165 PMC1282458

[78] U.S. Centers for Disease Control and Prevention (CDC). National Diabetes Prevention Program: PreventT2 Curriculum and Handouts 2024 [ https://www.cdc.gov/diabetes-prevention/php/lifestyle-change-resources/t2-curriculum.html#:~:text=The%20PreventT2%20curriculum%20is%20based,%2C%20physical%20activity%2C%20and%20diet

[79] Taylor SE. Social support: a review. Oxf Handb Health Psychol. 2011;1:189–214.

[80] Baucom KJ, Bauman T, Gutierrez Chavez M, Nemirovsky Y, Aguirre MC, Ramos C, et al. Barriers to participation and lifestyle change among lower versus higher income participants in the National Diabetes Prevention Program: lifestyle coach perspectives. Translational Behav Med. 2022;12(8):860–9. 10.1093/tbm/ibac032 PMC9385121 35554612

[81] Bishop J, Irby MB, Isom S, Blackwell CS, Vitolins MZ, Skelton JA. Diabetes prevention, weight loss, and social support: program participants’ perceived influence on the health behaviors of their social support system. Fam Community Health. 2013;36(2):158–71. 23455686 10.1097/FCH.0b013e318282b2d3 PMC3828626

[82] O’Neal LJ, Scarton L, Dhar B. Group social support facilitates adoption of healthier behaviors among black women in a community-initiated national diabetes prevention program. Health Promot Pract. 2022;23(6):916–9. 34628960 10.1177/15248399211045989

[83] McCurley JL, Gutierrez AP, Gallo LC. Diabetes prevention in US hispanic adults: a systematic review of culturally tailored interventions. Am J Prev Med. 2017;52(4):519–29. 27989451 10.1016/j.amepre.2016.10.028 PMC5362335

[84] Zare H, Delgado P, Spencer M, Thorpe RJ Jr, Thomas L, Gaskin DJ, et al. Using Community Health Workers to address barriers to Participation and Retention in Diabetes Prevention Program: a Concept Paper. J Prim Care Community Health. 2022;13:21501319221134563. 36331112 10.1177/21501319221134563 PMC9638527

[85] O’Mara-Eves A, Brunton G, Oliver S, Kavanagh J, Jamal F, Thomas J. The effectiveness of community engagement in public health interventions for disadvantaged groups: a meta-analysis. BMC Public Health. 2015;15:1–23. 25885588 10.1186/s12889-015-1352-y PMC4374501

[86] Gunn CM, Martinez LSS, Battaglia TA, Lobb R, Chassler D, Hakim D, et al. Integrating community engagement with implementation science to advance the measurement of translational science. J Clin Translational Sci. 2022;6(1):e107. 10.1017/cts.2022.433 PMC9549478 36285013

[87] Lythreatis S, Singh SK, El-Kassar A-N. The digital divide: a review and future research agenda. Technol Forecast Soc Chang. 2022;175:121359.

